# A Chinese Herbal Decoction, Dang Gui Bu Xue Tang, Prepared from Radix Astragali and Radix *Angelicae sinensis*, Ameliorates Insulin Resistance Induced by A High-Fructose Diet in Rats

**DOI:** 10.1093/ecam/nep004

**Published:** 2011-02-14

**Authors:** I-Min Liu, Thing-Fong Tzeng, Shorong-Shii Liou

**Affiliations:** ^1^Department of Pharmacy, Tajen University, Yanpu Shiang, Ping Tung Shien, Taiwan; ^2^Department of Internal Medicine, Pao Chien Hospital, Ping Tung City, ROC, Taiwan

## Abstract

Dang Gui Bu Xue Tang (DBT), a Chinese medicinal decoction contains Radix *Angelicae sinensis* (Danggui) and Radix Astragali (Huangqi) at a ratio of 1 : 5, is used commonly for treating women's ailments. This study was conducted to explore the effects of this preparation on insulin resistance in rats fed with 6-week diet containing 60% fructose. Similar to the action of rosiglitazone (4 mg kg^−1^ per day by an oral administration), repeated oral administration of DBT (2.5 g kg^−1^ per day) for 14 days was found to significantly alleviate the hyperglycemia but made no influence on plasma lipid profiles nor weight gain in fructose chow-fed rats. Also, the higher degree of insulin resistance as measured by homeostasis model assessment of basal insulin resistance in fructose chow-fed rats was significantly decreased by repeated DBT treatment. DBT displays the characteristic of rosiglitazone by increasing the whole-body insulin sensitivity in fructose chow-fed rats after 2-week treatment, as evidenced by the marked elevation of composite whole-body insulin sensitivity index during the oral glucose tolerance test. DBT improves insulin sensitivity through increased post-receptor insulin signaling mediated by enhancements in insulin receptor substrate-1-associated phosphatidylinositol 3-kinase step and glucose transporter subtype 4 translocation in soleus muscles of animals exhibiting insulin resistance. DBT is therefore proposed as potentially useful adjuvant therapy for patients with insulin resistance and/or the patients who wish to increase insulin sensitivity.

## 1. Introduction

Insulin resistance, a metabolic disorder raised seriously around the world, characterized by a diminished ability of insulin-sensitive tissues and a marked decrease of glucose metabolism in response to insulin resulting from complex interactions between genetic and environmental factors, is associated with some common diseases including type-2 diabetes, hypertension, obesity, and coronary heart disease [1]. The use of pharmacological intervention in addition to lifestyle modifications with weight loss and moderate exercise is accepted as a positive factor in health, and these are viewed as particularly useful in the management of obesity and cardiovascular diseases as well as prevention of insulin resistance and type-2 diabetes mellitus [2]. Thiazolidinediones (TZD), agonists of the peroxisome proliferator-activated receptor (PPAR)*γ*, enhance insulin sensitivity and improve metabolic control in patients with type-2 diabetes [3]. Despite the proven efficacy, a number of deleterious side effects of TZDs, including rosiglitazone and pioglitazone, have been noted such as weight gain and the documented aggravation of advanced heart failure through fluid retention [4]. Accordingly, efforts have been mounted to generate modulators that retain the beneficial clinical effects while avoiding these side effects. It seems, therefore, that development of new agents may be helpful in the therapy of diabetic patients with insulin resistance.

Dang Gui Bu Xue Tang (DBT), also known as Tangkuei and Astragalus Decoction, is widely employed in traditional Chinese medicine (TCM) because of the marked hematopoietic properties of this preparation [5]. In Mandarin, the herb *Angelica sinensis* is named as Dang Gui, Bu Xue refers to hematopoietic effects, and Tang is the term for solution. DBT was formulated originally during the Jin Dynasty and contained two main ingredients, Radix *Angelicae sinensis* (Danggui) and Radix Astragali (Huangqi) in a ratio of 1 : 5 [6, 7]. At this ingredient ratio, DBT has been found to reduce menopausal symptoms [8]. This formula treats consumptive fatigue and internal injury which causes deficient Qi and blood, and “floating yang” appears externally. Recent findings indicate that DBT has the ability to promote hematopoietic function, stimulate cardiovascular circulation, prevent osteoporosis and antagonize the activity of tumors [6, 9]. Actually, DBT has an ability to lower higher plasma glucose in streptozotocin-induced diabetic rats (STZ-diabetic rats), the type-1 diabetes-like animal model [7]. Additionally, DBT has been found to retard the progression of diabetic nephropathy through suppression of transforming growth factor-*β*_1_ gene expression in STZ-diabetic rats [7]. It seems that this traditional Chinese herbal preparation is valued in the glucose homeostasis, and may therefore utilize as adjuvant therapy for control of diabetes and its complications. However, little information is available regarding the effect of DBT on insulin resistance.

The consumption of fructose worldwide has largely increased due to an increased consumption of soft drinks and other beverages that are high in fructose, and due to the consumption of breakfast cereals, baked goods and prepared desserts sweetened with high-fructose corn syrup. Studies in rats have demonstrated that a high intake of fructose produced a decline of insulin sensitivity in the liver and peripheral tissues [10]. Hence, fructose has been implicated as a useful tool in inducing insulin resistance in animals. Thus, for this study, we employed rats with insulin resistance induced by the diet containing 60% fructose to investigate the effect of DBT on insulin resistance.

## 2. Methods

### 2.1. Materials

The water decocted concentrated powders for DBT (Cat. no KPC3570) was produced by Kaiser Pharmaceutical Co., Ltd (Tainan, Taiwan) under internationally certified good manufacturing practices guidelines. The product was registered (Code no 015648) by Committee on Chinese Medicine and Pharmacy, Department of Health, Executive Yuan, (Taiwan). The ratio of Radix *A. sinensis* (Danggui) and Radix Astragali (Huangqi) used in DBT is 1 : 5 [6, 7]. The experienced botanists and chemists of the supplier use macroscopic and microscopic examinations as well as thin-layer chromatography and high-performance liquid chromatography identification to authenticate the plants, plant parts used and process raw herbs. The reference specimens were deposited at the herbarium of the supplier. Standard rat chow containing 60% vegetable starch, 5% fat and 18% protein (Cat. no #2018) and fructose-rich rat chow containing 60% fructose, 5% fat and 18% protein (Cat. no #TD 89247) were obtained from Harlan Teklad (Madison, WI). Protein A-Sepharose beads were purchased from Sigma-Aldrich, Inc. (St Louis, MO). Rosiglitazone maleate (Avandia) was from GlaxoSmithKline (Research Triangle Park, NC). The diagnostic kits for determinations for plasma levels of glucose (Cat. no COD12503), cholesterol (Cat. no COD11539) and triglyceride (Cat. no COD11529) were purchased from BioSystem (Barcelona, Spain). Rat insulin enzyme-linked immunosorbent assay (ELISA) kit was obtained from Linco Research, Inc. (St Charles, MO; Cat. no #EZRMI-13K). The kit for protein assay was purchased from Bio-Rad Laboratories (CA, USA). Anti-insulin receptor (IR) *β*-subunit antibodies (Cat. no #MS-634 for immunoprecipitation; Cat. no #MS-636 for western blotting), anti-insulin receptor substrate (IRS)-1 antibody (Cat. no #MS-630) and anti-phosphotyrosine antibody (Cat. no #MS-445) were obtained from NeoMarkers (Fremont, CA, USA). Anti-phosphatidylinositol (PI) 3-kinase p85 antibody (Cat. no #4292), anti-Akt antibody (Cat. no #9272), anti-phospho-Ser^473^Akt antibody (Cat. no #9271), anti-phospho-Thr^308^Akt antibody (Cat. no #9275), anti-phospho-(Ser/Thr) Akt substrate antibody (Cat. no #9611) and anti-glucose transporter subtype 4 (GLUT 4) antibody (Cat. no #2299) were purchased from Cell Signaling Technology (Beverly, MA). Anti-AS160 (Rab GAP) antibody was obtained from Upstate (Charlottesville, VA; Cat. no #07-741). ECL Western Blotting Systems were obtained from Amersham Corp. (Braunschweig, Germany). Bovine insulin was obtained from Novo Nordisk (Bagsvaerd, Denmark). All other reagents were obtained from standard sources.

### 2.2. Animal Models

Male Wistar rats aged 8 weeks were obtained from the National Laboratory Animal Center (Taipei, Taiwan). They were maintained in a temperature-controlled room (25 ± 1°C) and kept on a 12 : 12 light-dark cycle (light on at 06:00 h) in our animal center. Food and water were available *ad libitum*. The rats were divided into two experimental groups. One group of rats was randomly assigned to receive the fructose chow for 6 additional weeks to induce insulin resistance [11]. The remaining group receiving standard chows during the 6-week period was designated as the control group. All animal procedures were performed according to the Guide for the Care and Use of Laboratory Animals of the National Institutes of Health, as well as the guidelines of the Animal Welfare Act.

The homeostasis model assessment of basal insulin resistance (HOMA-IR) was used to calculate an index from the product of the fasting concentrations of plasma glucose (mM) and plasma insulin (*μ*U mL^−1^) divided by 22.5 [12]. Lower HOMA-IR values indicated greater insulin sensitivity, whereas higher HOMA-IR values indicated lower insulin sensitivity (insulin resistance).

### 2.3. Treatment Protocols

The rats fed with 6-week fructose chow were used as insulin-resistant animals. Powders of DBT were dissolved in distilled water for oral administration at the desired doses (0.5, 1.5, 2.5 g kg^−1^ per day) in a volume of 10 mL kg^−1^ once a day into insulin-resistant rats. The effect of DBT was determined for 14 consecutive days in subsequent experiments. Another group of fructose chow-fed rats was treated similarly but with the same volume of vehicle (distilled water) as was used to dissolve DBT during the 2-week treatment period. Additionally, rosiglitazone was given by oral gavage (4 mg kg^−1^ per day) once a day for 14 days to the separate groups of fructose chow-fed rats. This dose was selected since it was comparable to the dose found to rapidly induce PPAR*γ*-dependent genes [13]. The rats were maintained on a fructose chow diet during the 2-week treatment period. Water was made available *ad libitum* throughout the experiment.

### 2.4. Oral Glucose Tolerance Test

Two weeks after DBT or rosiglitazone treatment, an oral glucose tolerance test (OGTT) was performed using an oral of glucose (1 g kg^−1^) for 2 h in fructose chow-fed rats. Animals were food-restricted and were given only water to drink throughout the night prior to the OGTT procedure. Plasma glucose and insulin concentrations were measured before (taken as 0 min) and 30, 60, 90 and 120 min after the glucose load.

Insulin sensitivity was calculated using the composite whole body insulin sensitivity index (ISIcomp) during the OGTT [14]. ISIcomp was estimated with the formula as following: ISIcomp = 10 000/square root of ((mean plasma insulin × mean plasma glucose during OGTT) × (fasting plasma glucose × fasting plasma insulin)).

### 2.5. Blood Sampling and Analysis

Blood sample of rats were collected from the lateral tail vein of animals anesthetized with sodium pentobarbital (30 mg kg^−1^) administered intraperitoneally (i.p.). Samples were centrifuged at 2000 g for 10 min at 4°C, plasma was removed and aliquot for the respective analytical determinations. Plasma glucose concentration was measured by glucose oxidase method (Hitachi 717 autoanalyzer; Hitachi Ltd, Tokyo, Japan). Levels of cholesterol and triglycerides in total plasma were analyzed enzymatically (Hitachi 717 autoanalyzer). The ELISA was employed to quantify the plasma insulin level using a commercially available kit. The test compounds used in this study did not affect the binding of peptide with antibodies. All samples were analyzed in triplicate.

### 2.6. In Vivo Insulin Receptor Activation

To assess DBT on insulin receptor activation *in vivo*, rats in the fed state were anesthetized with sodium pentobarbital at the end of the 2-week treatment period. Then, a bolus of insulin (10 U kg^−1^) was injected into portal vein of rats, as described previously [15]. Approximately 120 s after insulin injection, rats were sacrificed and the soleus muscle was immediately extirpated, washed with cold phosphate buffer and cut into 200–300 mg portions, which were then stored separately at −70°C for subsequent immunoprecipitation and immunoblot analyses.

### 2.7. Muscle Processing

Cytosol and membrane fractions were prepared according to the previous method [16]. Briefly, muscles used for measuring insulin signaling were weighed while still frozen and homogenized (Polytron, Brinkmann Instruments, Inc., Westbury, NY) in 0.4 mL homogenizing buffer containing 250 mM sucrose, 20 mM Tris (pH 7.5), 2 mM EDTA, 0.5 mM EGTA, 20 *μ*g mL^−1^ leupeptin, 10 *μ*g mL^−1^ aprotinin, 174.2 *μ*g mL^−1^ phenylmethylsulfonyl fluoride and 20 mM dithiothreitol. The homogenate was centrifuged at 100 000 g for 1 h at 4°C. The supernatant (cytosolic extract) was transferred to a tube kept on ice, whereas the pellet was resuspended in 0.45 mL homogenizing buffer containing 5% Triton X-100. The resuspended pellet fraction was then centrifuged at 14 000 g for 5 min at 4°C, and the pellet was discarded. The supernatant from this spin constitutes the membrane extract. Protein concentrations were determined by the BioRad protein dye binding assay. The supernatant was stored at −80°C until used in immunoprecipitation and western immunoblotting.

### 2.8. Immunoprecipitation

Muscle lysate (500 *μ*g) was subjected to immunoprecipitations with anti-IR *β*-subunit antibodies, anti-IRS-1 antibody, or anti-AS160 (Rab GAP) antibody at 4°C overnight, followed by shaking with protein A-Sepharose beads for 1 h. The bead-Protein A-antibody-antigen complexes were precipitated by brief centrifugation. The pellets were washed three times in ice-cold buffer (0.5% Triton X-100, 100 mM Tris, pH 7.4, 10 mM EDTA and 2 mM sodium vanadate), resuspended in Laemmli sample buffer and boiled for 5 min. The sepharose beads were precipitated by brief centrifugation and the supernatant prepared for sodium dodecyl sulfate-polyacrylamide gel electrophoresis (SDS-PAGE, 10% acrylamide gel) using a Bio-Rad Mini-Protein II system (55 and 130 V during the stacking and separation phases, resp.). Protein was transferred to a polyvinylidene difluoride (PVDF) membrane using the Bio-Rad Trans-Blot system (2 h at 20 V in 25 mM Tris, 192 mM glyceine and 20% MeOH). Following transfer, the membrane was probed with anti-IR *β*-subunit antibody, anti-IRS-1 antibody, anti-phosphotyrosine antibody, anti-p85 subunit of PI 3-kinase antibody or anti-AS160 antibody according to the manufacturers' instructions.

### 2.9. Immunoblotting

Equal amounts (50 *μ*g) of protein were prepared from muscle homogenates, subjected to SDS-PAGE, transferred to PVDF membrane as described above, and blotted with anti-PI 3 kinase p85 antibody, anti-Akt antibody and anti-GLUT 4 antibody, according to the manufacturer's instructions. Protein phosphorylation of Akt was measured using the anti-phospho-Ser^473^Akt antibody and anti-phospho-Thr^308^Akt antibody. Phosphorylation of Akt substrate of 160 kDa (PAS-AS160) was detected using the anti-phospho-(Ser/Thr) Akt substrate antibody. PAS recognizes Akt phosphorylation motif peptide sequences (RXRXXpT/S). After three 5 min washes in TBST (20 mM Tris-HCl (pH 7.5), 150 mM NaCl and 0.05% Tween 20), membranes were incubated with the appropriate peroxidase-conjugated secondary antibodies. The membranes were then washed three times in TBST and visualized on X-ray film using the enhanced chemiluminescence detection system. Densities of the obtained immunoblots were quantified using a laser densitometer. The mean value for samples from vehicle-treated standard chow-fed rats on each immunoblot, expressed in densitometry units, was adjusted to a value of 1.0. All experimental sample values were then expressed relative to this adjusted mean value.

### 2.10. Statistical Analysis

Data are expressed as the mean ± SD for each group of animals at the number (*n*) indicated in tables. Statistical differences among groups were determined by using two-way repeated-measures ANOVA. The Dunnett range *post-hoc* comparisons were used to determine the source of significant differences where appropriate. A *P*-value <.05 was considered statistically significant.

## 3. Results

### 3.1. General Characteristics of Rats

Animals maintained on the 6-week high-fructose diet exhibited significant hyperglycemia and hyperinsulinemia compared with the standard chow-fed group for the same feeding period (Table 1). Plasma glucose levels in fructose chow-fed rats were significantly decreased to near those observed in the standard chow-fed group after 2 weeks of rosiglitazone (4 mg kg^−1^ per day) treatment (Table 1). Following the administration of DBT at daily oral dosage of 2.5 g kg^−1^ for 14 consecutive days, plasma glucose levels in fructose chow-fed rats fell to a value significantly lower than that from their vehicle-treated counterparts (*P* < .05), showing a plasma glucose-lowering activity of 13.4 ± 3.1% (Table 1). DBT even at the higher daily oral dosage influences neither the plasma insulin levels nor body weight of fructose chow-fed rats. However, fructose chow-fed rats receiving rosiglitazone (4 mg kg^−1^ per day) treatment exhibited lower plasma insulin level and gained more body weight comparing with DBT-treated groups at the end of the 2-week treatment period (Table 1).

Plasma triglyceride level was significantly reduced in fructose chow-fed rats after 14 days of rosiglitazone (4 mg kg^−1^ per day; Table 1). Although the plasma triglyceride level in fructose chow-fed rats tended to be reduced by 14 days of DBT treatment, there was no significant difference between any treatments in fructose chow-fed rats group (Table 1). At the termination of 2-week treatment, the total plasma cholesterol in fructose chow-fed rats was reduced without significant differences between any treatment and those in standard chow-fed group.

The HOMA-IR score in rats feed with 6-week high-fructose diet was higher by 3.5-fold times that of the standard chow-fed group, which markedly fell to 47% of their vehicle-treated counterparts following 14 days of rosiglitazone (4 mg kg^−1^ per day) treatment (Table 1). The HOMA-IR score in fructose chow-fed rats receiving 2-week DBT (2.5 g kg^−1^ per day) treatment showed a decrease to 80% of the score observed in the vehicle-treated counterparts (Table 1).

### 3.2. Insulin Sensitivity

**Figure 1:**
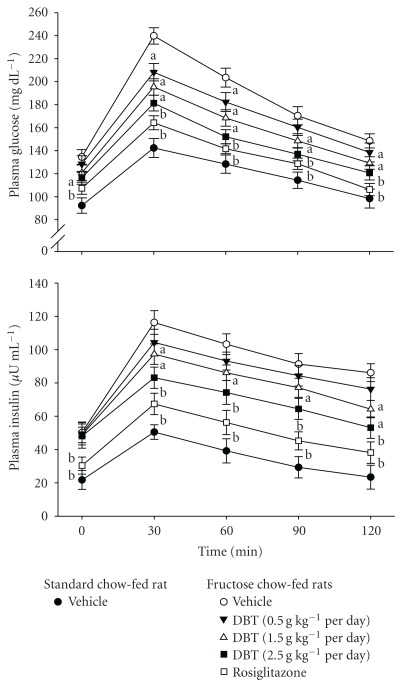
Plasma glucose and insulin responses during an oral glucose (1 g kg^−1^) tolerance test (OGTT) in fructose chow-fed rats repeatedly receiving oral administration of DBT at 0.5 g kg^−1^ (filled inverted triangle), 1.5 g kg^−1^ (open triangle) or 2.5 g kg^−1^ (filled square), once a day for 14 days. Rosiglitazone was given by oral gavage (4 mg kg^−1^ per day) for 14 days to the separate groups of the fructose chow-fed rats (open square). The vehicle (distilled water) used to dissolve the tested medications was given at the same volume. Values (mean ± SD) were obtained from each group of eight animals. ^a^ *P* < .05 and ^b^ *P* < .01 compared with the values of vehicle-treated fructose chow-fed rats (unfilled circle) at the indicated times, respectively.

Plasma glucose levels were significantly elevated during the OGTT in fructose chow-fed rats compared to the standard chow-fed group at all time points tested (Figure 1). In the vehicle-treated fructose chow-fed rats, plasma glucose concentrations increased from fasting levels of 134.5 ± 6.4 mg dL^−1^ to nearly 239.8 ± 7.1 mg dL^−1^ by 30 min and were still greatly increased over baseline levels 2 h after the oral glucose challenge. The fructose chow-fed rats treated with DBT for 2 weeks (2.5 g kg^−1^ per day) showed a significant elevation in plasma glucose concentrations at 30 min but returned to basal levels within 2 h after the oral glucose administration; similar results were obtained with rosiglitazone (4 mg kg^−1^ per day)-treated fructose chow-fed group (Figure 1).

Fasting plasma insulin concentrations were significantly greater in fructose chow-fed rats compared with the standard chow-fed group and remained higher at all time points throughout the OGTT study period (Figure 1). The plasma insulin concentrations of fructose chow-fed rats in both DBT- and rosiglitazone-treated groups increased 30 min after the oral glucose challenge, but returned to their respective fasting levels by 2 h after OGTT; the effect of DBT was more remarkable at the daily oral dosage at 2.5 g kg^−1^ (Figure 1).

The ISIcomp was also significantly lower in the fructose chow-fed group as compared to that in standard chow-fed group. Although not as effective as that produced by rosiglitazone (4 mg kg^−1^ per day), 2 weeks of DBT (2.5 g kg^−1^ per day) treatment increased ISIcomp of fructose chow-fed rats to 1.7-fold of the value in their vehicle-treated counterparts (Table 1).

### 3.3. The Protein Levels and the Degree of Insulin-Stimulated Phosphorylation of IR

**Figure 2:**
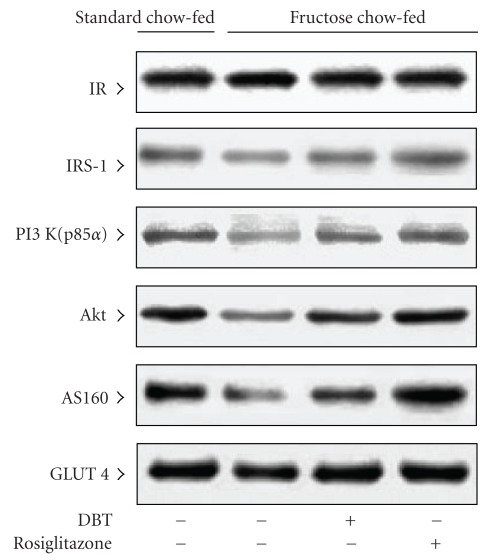
A Representative immunoblots of protein expression of insulin receptor-related signaling mediators in the soleus muscles of fructose chow-fed rats following repeated oral administration of DBT (2.5 g kg^−1^ per day) or rosiglitazone (4 mg kg^−1^ per day) for 14 days. Rats that did not receive any treatment were given the same volume of vehicle (distilled water) used to dissolve the test medications. Findings were reproduced on four separate occasions. Quantification of the data is shown in Table 2.

Following 2-week treatment, there was difference in the expression of IR protein in soleus muscle of rats between any groups; treatment with either rosiglitazone (4 mg kg^−1^ per day) or DBT (2.5 g kg^−1^ per day) did not modify these values (Figure 2, Table 2).

**Figure 3:**
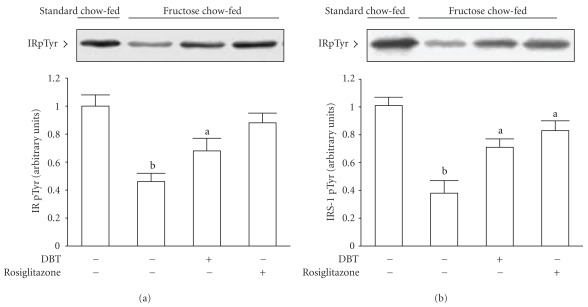
Representative immunoblots of insulin-stimulated phosphorylation of insulin receptor (a) and insulin receptor substrate-1 (b) in the soleus muscles of fructose chow-fed rats after repeated oral administration of DBT (2.5 g kg^−1^ per day) or rosiglitazone (4 mg kg^−1^ per day) for 14 days. Rats that did not receive any treatment were given the same volume of vehicle (distilled water) used to dissolve the test medications. Findings were reproduced on four separate occasions. Quantification of protein levels expressed as mean with SD (*n* = 5 per group) in each column. ^a^ *P* < .05 and ^b^ *P* < .01 represents the level of significance compared with the values with vehicle-treated standard chow-fed rats, respectively.

The degree of insulin-stimulated increment in tyrosine phosphorylation of IR in soleus muscle was clearly lowered in fructose chow-fed rats as relative to those from standard chow-fed group (Figure 3). Under insulin stimulation, 2-week rosiglitazone (4 mg kg^−1^ per day) treatment elevated the extent of tyrosine phosphorylation of IR in soleus muscle of fructose chow-fed rats to 1.9-fold of that from their vehicle-treated counterparts (Figure 3). Also, the degree of tyrosine phosphorylation of IR in soleus muscle of fructose chow-fed rats was ameliorated by 2-week DBT (2.5 g kg^−1^ per day) treatment (Figure 3).

### 3.4. The Protein Levels and the Degree of Insulin-Stimulated Phosphorylation of IRS-1

The expression of IRS-1 protein in soleus muscles of fructose chow-fed rats was close to 50% of that in standard chow-fed group (Figure 2, Table 2). At the end of the 2-week rosiglitazone (4 mg kg^−1^ per day) treatment, protein expression of IRS-1 in soleus muscles of fructose chow-fed rats resembled that of the standard chow-fed group (Figure 2, Table 2). Two-week DBT (2.5 g kg^−1^ per day) treatment also raised IRS-1 protein expression in soleus muscles of fructose chow-fed rats, but did not achieve the level in standard chow-fed rat (Figure 2, Table 2).

The insulin-stimulated IRS-1 tyrosine phosphorylation in soleus muscles of fructose chow-fed rats following rosiglitazone treatment was returned to levels comparable to those of standard chow-fed animals (Figure 3, Table 2). Two weeks treatment of fructose chow-fed rats with DBT (2.5 g kg^−1^ per day) recovered the insulin-stimulated IRS-1 tyrosine phosphorylation in soleus muscles to levels close those of standard chow-fed animals (Figure 3, Table 2).

### 3.5. The Protein Levels and Activity of PI 3-Kinase

The basal level of p85 regulatory subunit of PI 3-kinase in soleus muscle of fructose chow-fed rats was depressed to nearly 48% of that in standard chow-fed group. The expression of p85 regulatory subunit of PI 3-kinase in soleus muscles was increased after fructose chow-fed animals receiving 2 weeks of rosiglitazone (4 mg kg^−1^ per day) or DBT (2.5 g kg^−1^ per day; Figure 2, Table 2).

**Figure 4:**
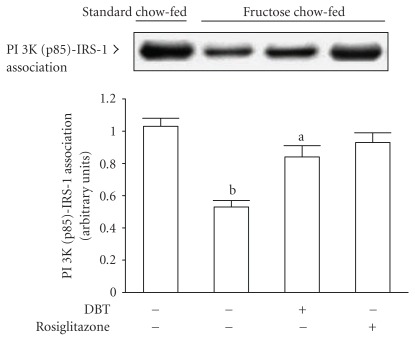
Representative immunoblots of the PI 3-kinase associated with IRS-1 in insulin-stimulated soleus muscles of fructose chow-fed rats receiving oral administration of DBT (2.5 g kg^−1^ per day) or rosiglitazone (4 mg kg^−1^ per day) for 14 days. Rats that did not receive any treatment were given the same volume of vehicle (distilled water) used to dissolve the test medications. Findings were reproduced on four separate occasions. Quantification of protein levels expressed as mean with SD (*n* = 5 per group) in each column. ^a^ *P* < .05 and ^b^ *P* < .01 represents the level of significance compared to the values with vehicle-treated standard chow-fed rats, respectively.

Two-week treatment with rosiglitazone (4 mg kg^−1^ per day) significantly improved the insulin-stimulated state of PI 3-kinase associated with IRS-1 in soleus muscles of fructose chow-fed rats (Figure 4). Although the degree of insulin-stimulated association between PI 3-kinase and IRS-1 in soleus muscle of DBT-treated fructose chow-fed rats was still lowered in relation to values from those of standard chow-fed animals, which was increased to 1.6-fold of that from their vehicle-treated counterparts (Figure 4).

### 3.6. The Protein Levels and the Degree in Insulin-Stimulated Phosphorylation of Akt

The expression of Akt protein in soleus muscles of fructose chow-fed rats was lower than that in standard chow-fed group (Figure 2, Table 2). After 2 weeks of rosiglitazone (4 mg kg^−1^ per day) treatment, the Akt protein expression in soleus muscle of fructose chow-fed rats was returned to that for standard chow-fed group (Figure 2, Table 2). It observed that 2-week DBT (2.5 g kg^−1^ per day) treatment elevated Akt protein level in soleus muscles of fructose chow-fed rats to 1.5-fold of that from their vehicle-treated counterparts (Figure 2, Table 2).

**Figure 5:**
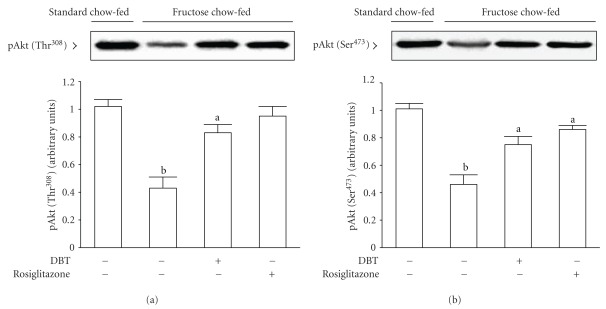
Representative immunoblots of insulin-stimulated phosphorylation of Akt on both Thr^308^ (a) and Ser^473^ (b) in soleus muscles of fructose chow-fed rats receiving oral administration of DBT (2.5 g kg^−1^ per day) or rosiglitazone (4 mg kg^−1^ per day) for 14 days. Rats that did not receive any treatment were given the same volume of vehicle (distilled water) used to dissolve the test medications. Findings were reproduced on four separate occasions. Quantification of protein levels expressed as mean with SD (*n* = 5 per group) in each column. ^a^ *P* < .05 and ^b^ *P* < .01 represents the level of significance compared with the values with vehicle-treated standard chow-fed rats, respectively.

Insulin-stimulated phosphorylation of Akt on both Thr^308^ and Ser^473^ in soleus muscles of fructose chow-fed rats receiving 2 weeks of rosiglitazone (4 mg kg^−1^ per day) treatment were increased to nearly 2-fold of those from their vehicle-treated counterparts (Figure 5). Two-week DBT (2.5 g kg^−1^ per day) treatment also enhanced the phosphorylation degree of Akt tyrosine (Thr^308^) and Akt serine (Ser^473^) in soleus muscles of fructose chow-fed animals in response to insulin-stimulation (Figure 5).

### 3.7. The Protein Levels and the Degree in Insulin-Stimulated Phosphorylation of AS160

The protein content of AS160 in soleus muscle of fructose chow-fed rats was depressed to 40% of that in standard chow-fed group; 2-week treatment with rosiglitazone (4 mg kg^−1^ per day) elevated AS160 expression in soleus muscle of fructose chow-fed rats close that of standard chow-fed rats (Figure 2, Table 2). The depressed protein level of AS160 in soleus muscles of fructose chow-fed rats was increased after 2-week DBT (2.5 g kg^−1^ per day) treatment although not to the levels of standard chow-fed group (Figure 2, Table 2).

**Figure 6:**
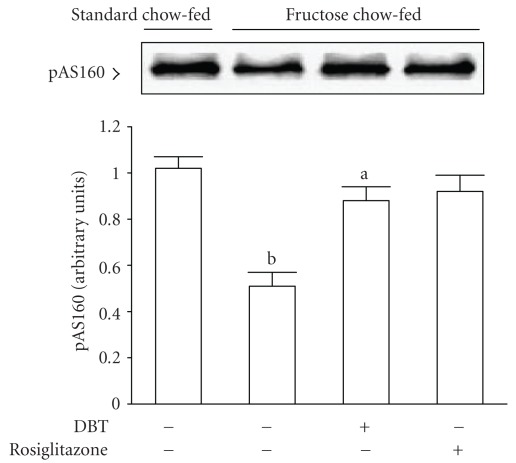
Representative immunoblots of insulin-stimulated phosphorylation of AS160 in the soleus muscles of fructose chow-fed rats receiving oral administration of DBT (2.5 g kg^−1^ per day) or rosiglitazone (4 mg kg^−1^ per day) for 14 days. Rats that did not receive any treatment were given the same volume of vehicle (distilled water) used to dissolve the test medications. Findings were reproduced on four separate occasions. Quantification of protein levels expressed as mean with SD (*n* = 5 per group) in each column. ^a^ *P* < .05 and ^b^ *P* < .01 represents the level of significance compared with the values with vehicle-treated standard chow-fed rats, respectively.

The phosphorylation of AS160 induced by insulin was markedly elevated in soleus muscles of fructose chow-fed rats receiving 2 weeks of rosiglitazone (4 mg kg^−1^ per day; Figure 6). Two-week DBT (2.5 g kg^−1^ per day) treatment also raised the degree of phosphorylation of AS160 in soleus muscles of fructose chow-fed rat in response to insulin stimulation (Figure 6).

### 3.8. The Protein Levels and Insulin-Stimulated Translocation of GLUT 4

Although the total GLUT 4 protein in soleus muscles of fructose chow-fed rats was lower than that in standard chow-fed group, there was no significant difference (Figure 2). The expression of total GLUT 4 protein in soleus muscles was raised in fructose chow-fed rats after 2 weeks of treatment, but the changes induced by neither rosiglitazone nor DBT was different from those of their vehicle-treated counterparts (Figure 2).

**Figure 7:**
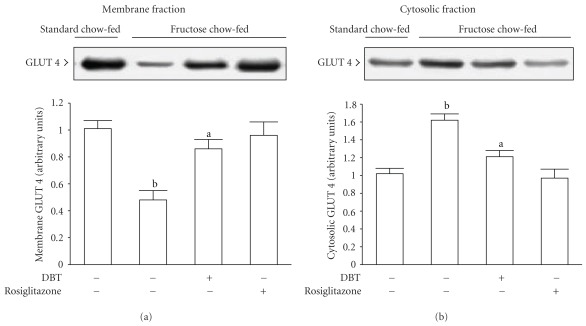
The autoradiograph resulting from Western blot analysis of representative protein levels for glucose transporter subtype 4 (GLUT 4) in the membrane (a) and cytosolic (b) fractions of soleus muscle from fructose chow-fed rats responded to insulin stimulation following repeatedly oral administration of DBT (2.5 g kg^−1^ per day) or rosiglitazone (4 mg kg^−1^ per day) for 14 days. Rats that did not receive any treatment were given the same volume of vehicle (distilled water) used to dissolve the test medications. Similar results were obtained with an additional four replications. Quantification of protein levels expressed as mean with SD (*n* = 5 per group) in each column. ^a^ *P* < .05 and ^b^ *P* < .01 represents the level of significance compared to the values with vehicle-treated standard chow-fed rats, respectively.

Insulin-stimulated GLUT 4 protein expression in the membrane fraction of soleus muscle from fructose chow-fed rats was only about 30% of that in standard chow-fed rats; conversely, GLUT 4 protein content in the cytosolic fraction of the same sample was about 1.6-fold of that observed in the standard chow-fed group (Figure 7). In fructose chow-fed animals receiving 2-week rosiglitazone (4 mg kg^−1^ per day) treatment, insulin-stimulated GLUT 4 protein expression in the membrane fraction of soleus muscles was increased to achieve the level as in the standard chow-fed group, but the GLUT 4 protein level in the cytosolic fraction of same sample was at 60% of the level of their vehicle-treated counterparts (Figure 7). At the termination of 2-week DBT (2.5 g kg^−1^ per day) treatment, insulin-stimulated GLUT 4 protein expression in the membrane fraction of soleus muscles from fructose chow-fed animals increased to about 1.8-fold of those from the level of their vehicle-treated counterparts, whereas the amount of GLUT 4 protein in the cytosolic fraction of the soleus muscles from fructose chow-fed animals was changed to 70% of that observed in their vehicle-treated counterparts (Figure 7).

## 4. Discussion

Increasing consumption of dietary fructose could be one of the factors responsible for the development of obesity and the accompanying insulin resistance syndrome [10]. It has been established that insulin resistance, impaired glucose tolerance, hyperinsulinemia, hypertension and hyperlipidemia are associated with fructose intake in animal models [10]. The duration of high fructose feeding and the lipid content and type of the diets lead to discrepancies in the degree of insulin resistance and in the metabolic parameters. In this study, an increase in plasma glucose level associated with hyperinsulinaemia definitively suggests impaired insulin action on glucose regulation in 6-week fructose chow-fed rats. Two-week treatment regimen with DBT was found to significantly decrease the high plasma glucose concentration, but in contrast to the effect of rosiglitazone made less effect on the modification of dyslipidemia in fructose-induced insulin resistance. It seems that the major action of DBT in insulin-resistant rats might not be associated with controlling in synthesis and/or metabolism of lipid, regulation of the insulin-glucose axis represents an obvious factor in the assessment of the mechanism(s).

The degree of insulin resistance was found to be higher in rats fed with 6-week fructose chow as indicated by a higher HOMA-IR [12]. Compared to HOMA-IR index based on measurements of basal glucose and insulin, the ISIcomp provides a reasonable approximation of whole-body insulin sensitivity that represents a composite of hepatic and peripheral tissues and takes into consideration insulin sensitivity both in the basal state and after the ingestion of a glucose load [12, 14]. Although the reduced HOMA-IR value could support the fact that insulin resistance in fructose chow-fed rats was ameliorated by 14-day DBT treatment, DBT was found to increase whole-body insulin sensitivity in fructose chow-fed rats as evidenced by the marked elevation of ISIcomp in these animals after 2-week administration, as did rosiglitazone treatment. Clearly, these results imply that DBT has the ability to reverse the impaired responsiveness to insulin in insulin-resistant rats, demonstrating the insulin-sensitizing effect of the formula.

Skeletal muscle insulin resistance is a salient feature of type-2 diabetes. In humans and other mammals, skeletal muscle normally accounts for about 75% of whole body insulin-stimulated glucose transport [17]. Impaired ability of the muscle to respond to insulin is therefore disruptive to systemic glucose homeostasis. It has been documented that insulin action on glucose uptake and metabolism is much greater in skeletal muscle composed primarily of oxidative fibers (e.g., the soleus) as compared with glycolytic fibers (e.g., the epitrochlearis and extensor digitorum longus), even though the soleus muscle represents a small portion of the total muscle mass [18]. Actually, the increase in insulin action on skeletal muscle is likely to be related to increased protein expression-and/or functional activity of several key components of the insulin signal transduction cascade. Defects in the insulin signaling cascade leading to impaired glucose utilization are believed to play a key role in the pathogenesis of insulin resistance [19]. It is conceivable that IRS-1 tyrosine phosphorylation in response to insulin stimulation generally increases the association of IRS-1 with the PI 3-kinase, resulting in increased PI 3-kinase activity, which in turn leads to activation of serine/threonine kinase protein B (PKB or Akt) and, ultimately, to an enhancement in insulin-stimulated glucose disposal [20]. Therefore, we proposed that any observed increases in IRS-1 related signals in soleus muscles of fructose chow-fed rats after DBT treatment would provide strong evidence for the beneficial effects on the amelioration of impaired insulin action. With this aim in mind, soleus muscle samples were prepared from all animals after insulin stimulation.

Similar to the effect of rosiglitazone treatment, the impaired insulin action on IRS-1 tyrosine phosphorylation or IRS-1-associated PI 3-kinase activity in soleus muscle of fructose chow-fed rats has been improved by repeated treatment with DBT. Actually, phosphorylation of Akt on both Thr^308^ and Ser^473^ is required for maximal activation [21]. Two-week DBT treatment was found to significantly increase the protein levels of Akt accompanied to elevate insulin action on phosphorylation of Akt on both Thr^308^ and Ser^473^ in soleus muscles of fructose chow-fed rats. Here we show that the DBT-mediated recovery of insulin action was related to the improvement in IRS-1/PI 3-kinase/Akt signaling pathway in insulin-resistant soleus muscle. It seems possible that the effect of DBT on the improvement of post-receptor insulin signaling induced the subsequent increase in insulin sensitivity.

Considering that the regulation of glucose uptake into muscle cells via GLUT 4 is a fundamental action of insulin, and this process is impaired in type-2 diabetes [22], we examined the expression and translocation of GLUT 4 protein in soleus muscle. It was found that the protein levels of total GLUT 4 were not significantly reduced in soleus muscle of fructose chow-fed rats as relative to the normal control rats; this is consistent with the previous study demonstrating that alterations in GLUT 4 expression are not a primary cause for the development of insulin resistance [22]. Our data shows that rosiglitazone or DBT had no significant effect on whole-muscle expression of GLUT 4 protein in fructose chow-fed rats, but there was a definite improvement in the defective insulin action on GLUT 4 translocation from intracellular vesicles to the plasma membrane of soleus muscle given in fructose chow-fed rats by DBT treatment. Although the effect of DBT on the translocation of GLUT 4 protein in insulin-resistant soleus muscle was not as effective as that produced by rosiglitazone, based on the above findings, we considered that the effect of DBT on improvement in insulin sensitivity may be attributable to restored insulin signaling cascade involved in the regulation of the recruitment of GLUT 4 transporters from intracellular reserves and their subsequent insertion into the plasma membrane, thereby leading to increase glucose delivery into skeletal muscle.

There is considerable evidence that in response to insulin, phosphorylation of a recently identified protein, known as Akt substrate of 160 kDa (AS160), correlated significantly with IRS-1 and Akt phosphorylation and associated with accelerated rates of GLUT 4 translocation, such that GLUT 4 manifests predominantly at the cell surface and enhances insulin-stimulated glucose transport [23]. Similar to the effect of rosiglitazone, DBT was found to increase insulin-stimulated AS160 phosphorylation on PAS motifs in soleus muscles of fructose chow-fed rats. It seems that not only the impaired insulin-mediated phosphorylation, but also the defect in the protein expression involved in the IRS-1/PI 3-kinase/Akt/AS160 arm of the insulin signaling pathway was improved in soleus muscle of 2-week DBT-treated fructose chow-fed rats. It raised the possibility that the DBT-mediated action on the improvement of insulin sensitivity in fructose chow-fed rats may be associated with a point of the post-receptor insulin signaling for increasing cell surface GLUT 4 and glucose transport. It has been documented that rosiglitazone can sensitize AMP-activated protein kinase (AMPK) mediated glucose disposal in peripheral tissues under insulin-resistant states [24]. Although further studies are needed to determine whether the insulin-sensitizing effect of DBT is mediated through the action of the AMPK pathway, our findings provide a new insight to the pharmacological benefits of DBT, which displays the characteristics of rosiglitazone, retaining its insulin sensitization potential but, unlike rosiglitazone, does not cause any increase in body weight. Administration of DBT may be a suitable therapeutic adjunct for the treatment of insulin resistant patients and/or the patients who are particularly sensitive to the common TZD-induced side effects of weight gain and edema.

Following the most general principles of TCM, diabetes affects a patient's “qi (energy/life-force)” and cause weakness in circulatory system function. In TCM, the spleen is a source of vital energy and blood and a controller of blood circulation. When spleen qi is weak, its blood controlling function is disturbed; furthermore, the production of blood and qi are decreased. Treatment of diabetes by the principles of TCM therefore emphasizes improvements of “qi” and circulatory function [25]. In view of the findings presented in this report, the preparation known as DBT possesses the potential to accomplish these objectives. Most TCM remedies are prepared as solutions and formulated to contain different herbs in combination. The ratio of Radix *A. sinensis* and Radix Astragali used in DBT should be 1 : 5 as described in China in 1247 AD; however, the rationale of this formula has not been given. It has been documented that higher amounts of Radix *A. sinensis*-derived ferulic acid, and Radix Astragali-derived astragaloside IV, calycosin, and formononetin were found in DBT with Radix *A. sinensis* and Radix Astragali in 1 : 5 ratio [26]. Further studies will be required to identify the ingredients and/or identified chemicals in DBT that is responsible for the beneficial effects observed in the present study.

It should be noted that the scientific basis for the therapeutic effects of TCM, which utilizes herbs for therapy under the guidance of traditional theory, has not been established in the literature. The findings of the present study are of merit in revealing, for the first time, that DBT possesses the potential to reverse the inability of insulin to act on soleus muscle of rats receiving fructose-rich chow. The beneficial effects of DBT on improvements in whole-body insulin sensitivity are associated with amelioration of defective insulin action on specific post-receptor insulin signaling related to IRS-1-associated PI 3-kinase step and GLUT 4 translocation. This preparation may therefore prove useful as adjuvant therapy in the treatment of diabetes.

##  Funding

This study was supported by a grant from the National Science Council (NSC 95-2320-B-127-003-MY2) of Taiwan.

## Figures and Tables

**Table 1:** General characteristics of fructose chow-fed rats after 2 weeks of treatment.

Characteristics	Standard chow-fed	Fructose chow-fed				
	Vehicle	Vehicle	DBT (g kg^−1^ per day)			Rosiglitazone
			0.5	1.5	2.5	
Body weight (g/rat)	248.7 ± 9.8	266.8 ± 8.7	263.4 ± 8.4	258.5 ± 9.6	260.9 ± 9.4	302.3 ± 10.1^a,c^
Plasma glucose (mg dL^−1^)	92.3 ± 6.1^d^	134.5 ± 7.2^b^	128.4 ± 6.4^b^	120.7 ± 5.8^b^	116.4 ± 5.1^a,c^	106.3 ± 5.6^a,c^
Plasma insulin (*μ*U mL^−1^)	21.8 ± 5.3^d^	50.3 ± 4.7^b^	49.5 ± 5.4^b^	48.8 ± 5.6^b^	48.1 ± 4.9^b^	30.4 ± 5.8^d^
Plasma triglyceride (mg dL^−1^)	97.4 ± 9.4^d^	325.2 ± 12.1^b^	319.7 ± 10.7^b^	312.3 ± 11.8^b^	308.6 ± 12.4^b^	174.2 ± 10.5^b,d^
Plasma cholesterol (mg dL^−1^)	85.4 ± 6.3	90.6 ± 5.9	86.5 ± 5.1	84.2 ± 6.7	83.4 ± 5.3	78.3 ± 4.7
HOMA-IR score	4.9 ± 0.7^d^	16.7 ± 1.3^b^	15.6 ± 0.9^b^	14.5 ± 1.0^b^	13.8 ± 1.1^b^	7.9 ± 0.8^b,c^
ISIcomp	3.63 ± 0.18^d^	0.92 ± 0.14^b^	1.12 ± 0.19^b^	1.32 ± 0.15^b,c^	1.53 ± 0.17^b,c^	2.21 ± 0.15^a,c^

DBT was dissolved in distilled water for oral administration at the desired doses in a volume of 10 mL kg^−1^ once a day into rats receiving 6 weeks of fructose chow feeding. Rosiglitazone was also given by oral gavage (4 mg kg^−1^ per day) to the separate groups of the fructose chow-fed rats. The vehicle (distilled water) used to dissolve the tested medications was given at the same volume. ISIcomp was calculated after a 2 h OGTT. Values (mean ± SD) were obtained from seven rats. ^a^ *P* < .05 and ^b^ *P* < .01 compared with the values of vehicle-treated standard chow-fed rats in each group, respectively. ^c^ *P* < .05 and ^d^ *P* < .01 compared with the values of vehicle-treated fructose chow-fed rats in each group, respectively.

**Table 2:** Quantification of the specific insulin signaling proteins in soleus muscles of fructose chow-fed rats after 14-day treatment with DBT or rosiglitazone.

Relative units	Standard chow-fed	Fructose chow-fed		
	Vehicle	Vehicle	DBT (2.5 g kg^−1^ per day)	Rosiglitazone (4 mg kg^−1^ per day)
IR	1.00 ± 0.06	1.01 ± 0.06	0.98 ± 0.09	1.01 ± 0.07
IRS-1	1.01 ± 0.05^d^	0.48 ± 0.07^b^	0.78 ± 0.08^a,c^	0.92 ± 0.06^d^
PI 3 K (p85)	1.03 ± 0.06^d^	0.49 ± 0.05^b^	0.81 ± 0.06^a,d^	0.94 ± 0.05^d^
Akt	1.02 ± 0.04^d^	0.43 ± 0.06^b^	0.76 ± 0.05^a,c^	0.93 ± 0.08^d^
AS160	1.01 ± 0.05^d^	0.41 ± 0.07^b^	0.68 ± 0.04^a,c^	0.96 ± 0.09^d^
GLUT 4	1.00 ± 0.08^d^	0.89 ± 0.09	0.95 ± 0.07	0.97 ± 0.06

DBT or rosiglitazone was dissolved in distilled water for oral administration at the desired doses in a volume of 10 mL kg^−1^ once a day into rats receiving 6 weeks of fructose chow feeding. The vehicle (distilled water) used to dissolve the tested medications was given at the same volume. Values (mean ± SD) were obtained from each group of five animals. ^a^ *P* < .05 and ^b^ *P* < .01 compared with the values of vehicle-treated standard chow-fed rats in each group, respectively. ^c^ *P* < .05 and ^d^ *P* < .01 compared with the values of vehicle-treated fructose chow-fed rats in each group, respectively.
